# Knowledge, Attitude and Practices Towards Cervical Cancer and its Screening Among Women from Tribal Population: a Community-Based Study from Southern India

**DOI:** 10.1007/s40615-020-00760-4

**Published:** 2020-04-24

**Authors:** Supriti Ghosh, Sneha D. Mallya, Ranjitha S. Shetty, Sanjay M. Pattanshetty, Deeksha Pandey, Shama Prasada Kabekkodu, Kapaettu Satyamoorthy, Veena G. Kamath

**Affiliations:** 1 Department of Cell and Molecular Biology, Manipal School of Life Sciences, Manipal Academy of Higher Education, Manipal, Karnataka 576104 India; 2 Department of Community Medicine, Kasturba Medical College, Manipal Academy of Higher Education, Manipal, Karnataka 576104 India; 3 Centre for Indigenous Population, Kasturba Medical College, Manipal Academy of Higher Education, Manipal, Karnataka 576104 India; 4 Department of Health Policy, Prasanna School of Public Health, Manipal Academy of Higher Education, Manipal, Karnataka 576104 India; 5 Department of Obstetrics and Gynaecology, Kasturba Medical College, Manipal Academy of Higher Education, Manipal, Karnataka 576104 India

**Keywords:** Cervical cancer, Reproductive health, Sexual health, Information, Universal access, Tribal women

## Abstract

**Background:**

Cervical cancer continues to be a leading cancer among women in India. Despite availability of various screening techniques, majority of Indian women remain unscreened for cancer cervix. The increasing incidence could be attributed to the lack of awareness regarding cervical cancer screening and paucity of organized screening facilities in the country. This study assessed the knowledge, attitude and practices (KAP) towards cervical cancer screening among tribal women of coastal Karnataka, southern India.

**Methods:**

A community-based cross-sectional study was conducted among 1140 women aged 20–65 years from three tribes. Information on their KAP towards cervical cancer screening was collected using a semi-structured questionnaire.

**Results:**

Mean age of the participants was 39.8 ± 10.1 years. Although 82.9% of the participants reported to have heard of cervical cancer, 51% were aware that the disease could be prevented, and only 2.3% knew that it can be detected at an early stage. Over 75% of the participants did not have adequate knowledge regarding cervical cancer. However, majority of them (99.9%) had a favourable attitude towards cervical cancer screening. None of them had undergone cervical cancer screening prior to the study. The knowledge scores were significantly associated with age group, marital status, education level, socioeconomic status and tribal community of the participants (*p* < 0.05).

**Conclusion:**

Overall knowledge regarding cervical cancer among the surveyed women was poor, though they exhibited a positive attitude. This calls for a sustained health education and screening program to create awareness and improve the uptake of cervical cancer screening among these women**.**

## Introduction

The tribal population is an integral part of India’s social diversity and has the second largest concentration after that of the African continent. They account for 8.6% of the total population of the country as per the 2011 census [1]. Among these, 75 tribes have been classified as particularly vulnerable tribal groups (PVTGs) based on pre-agriculture level of technology, stagnant or declining population, extremely low literacy and subsistence level of economy [2]. In Karnataka, Scheduled Tribes (ST) account for 6.95% (4.24 million) of the total state population [3]. Of the two PVTGs identified in the state of Karnataka, Koraga tribe predominantly inhabits Udupi and Dakshina Kannada districts located in the southern part of the state [2]. Marathi Naika and Malekudiya are the other two tribes residing in these districts.

Poverty, illiteracy, meagre living conditions, lack of personal hygiene and poor health-seeking behaviour are known to be highly prevalent among these tribes. Further, early age at marriage, consanguinity, high parity and other behavioural factors among women in these tribal communities make them susceptible to sexually transmitted infections (STIs) [4–8].

Cancer cervix is the second most common cancer among women aged 15 to 44 years in India with an annual estimated incidence of 122,844 cases and mortality of 67,477 women [9]. Developing countries contribute to about 85%, and India alone accounts for one fourth of the world’s cervical cancer burden [10]. This is probably due to unavailability of nationwide regular cancer screening programs, lack of awareness and unwillingness of rural Indian women towards screening procedures for cancer cervix [11]. Being a part of tribal community, these aforementioned factors are expected to be widely prevalent among their women. In this direction, assessing their awareness regarding cervical cancer and its prevention is crucial. As there is paucity of such data from these tribes, especially Koraga women, the present study was undertaken with an aim to study their knowledge, attitude and practices towards cervical cancer, its causes, preventive measures and screening methods.

## Methods

This community-based cross-sectional study was conducted from July 2014 to June 2017 among 1140 married women from Koraga, Malekudiya and Marathi Naika tribes of Udupi district, Karnataka, India. As a part of a study which included screening for cancer cervix and detection of cervical DNA virus infections among tribal women in this region, their knowledge, attitude and practices (KAP) towards cervical cancer were assessed [12]. The study team performed a door-to-door survey of the eligible tribal women. Upon obtaining written informed consent from all the willing participants, a pre-designed semi-structured questionnaire was administered to collect baseline demographic information and questions pertaining to knowledge, attitude and practice towards cervical cancer.

Eleven knowledge-related components were considered to calculate the cumulative knowledge score. These questions included knowledge about any risk factor of cervical cancer, preventive measure and early detection of the disease. The risk factor options included viral infection, early age at first sexual intercourse, multiple sexual partners, high parity, use of oral contraceptives, smoking, poor genital hygiene and condom use. Scoring for each correct answer was given + 1, incorrect answer was – 1, and “do not know” was scored 0. Considering the distribution of knowledge scores among the study participants, cumulative score of 4 and above was categorized as adequate knowledge. Similar approach was undertaken to determine the cumulative attitude score. Three questions were considered for determining the attitude scores, viz. is cervical cancer screening important, should regular screening be made available at local health centres and willingness to be educated more about cervical cancer. Women with cumulative attitude score of 2 and above were considered to have favourable attitude towards cervical cancer screening. Women who had undergone Pap smear tests prior to the study were considered to follow fair practice towards cervical cancer screening.

Statistical Package for Social Sciences (SPSS) version 16 was used for data entry and analysis. Categorical data have been presented in frequencies and proportions. Univariable and multivariable logistic regressions were performed to estimate the strength of associations between knowledge score and socio-demographic variables of the participants and expressed as odds ratio (OR) and adjusted OR (AOR) with corresponding 95% confidence interval (CI). *p* value < 0.05 was considered statistically significant.

## Results

The study comprised of 1140 women from three tribal communities aged between 20 and 65 years. Mean age of the participants was 39.8 years (SD ± 10.1) with the majority of them being currently married (89.3%). About 34% of the participants were illiterates, and only 5.1% had more than 10 years of schooling. Of the participants who were currently employed, 39.8% were engaged in unskilled type of work which included predominantly manual labour. Nearly two third of the study participants (61.1%) were from low socio-economic status, as determined by the modified Udai Pareek scale [13]. A total of 39.6% of the study participants belonged to Koraga and Malekudiya tribal communities, while the rest of them were from Marathi Naika community.

About 945 (82.9%) study participants had heard about the term “cervical cancer,” and hence, only they could answer the subsequent questions regarding cervical cancer. Their knowledge and attitude about cervical cancer and screening are summarized in Table 1. Of these 945 women, 51.0% reported to know that the disease is preventable, though majority (97.7%) were ignorant about the fact that cervical cancer can be detected at an early stage. Only 7.0% of the participants knew about viral infection being a risk factor for the development of cervical cancer, and about 3.6% of the participants had a misconception that use of condom causes cervical cancer. A total of 19 (2.0%) women said Papanicolaou (Pap) test could be used for detection of cervical cancer, while only one participant mentioned biopsy as a detection tool. Source of information regarding cervical cancer claimed to be was mostly through relatives and friends (97.1%), while print and electronic media (TV, radio, newspaper) contributed to about 65.8%, and medical and health professionals contributed to a little over 32% of the information. However, it was positive to note that over 95% of the participants considered cervical cancer screening tests to be important and agreed that regular screening services should be made available in the health centres which cater to rural and remote areas. They also wanted to be educated more about cervical cancer by the healthcare professionals.

**Table 1:** Knowledge and attitude of the study participants about cervical cancer and screening (*n* = 945)

	Frequency (%)
Knowledge-related components	
Risk factors for development of cervical cancer	
Poor genital hygiene	361 (38.2)
Early age at sexual intercourse	271 (26.7)
Smoking	157 (16.6)
High parity	111 (11.6)
Use of oral contraceptives	81 (8.6)
Multiple sexual partners	69 (7.3)
Viral infection	66 (7.0)
Condom use	34 (3.6)
Do not know	467 (49.4)
Whether cervical cancer prevention is possible	
Yes	482 (51.0)
No	455 (48.1)
Do not know	8 (0.8)
Whether detection of this disease is possible at an early stage	
Do not know	923 (97.7)
Yes	22 (2.3)
Source of information	
Relatives or friends	918 (97.1)
Mass media	622 (65.8)
Health workers	217 (23.0)
Doctor	52 (5.5)
Health education	40 (4.2)
Attitude-related components	
Whether screening test for cervical cancer is important	
Yes	906 (95.9)
Not sure	39 (4.1)
Whether screening services should be made available in the rural health centres	
Yes	943 (99.8)
Not sure	2 (0.2)

Table 2 shows the knowledge, attitude and practice scores of the participants. About 77% of the participants had inadequate knowledge regarding cervical cancer prevention and screening. While almost everyone (99.1%) had a favourable attitude towards getting screened for cervical cancer if the services are made available, none of them had undergone any screening tests for detection of pre-malignant/malignant lesions of the cervix in the past.

**Table 2:** Knowledge, attitude and practice score of the participants (*n* = 945)

Items	Values	*p* value	95% CI
Knowledge score			
Adequate	212 (22.4)	< 0.0001	50.98–58.51
Inadequate	733 (77.6)		
Mean score	1.70 ± 1.89		
Range of score	0–7		
Attitude score			
Favourable	944 (99.9)	< 0.0001	99.11–99.91
Unfavourable	1 (0.1)		
Mean score	2.96 ± 0.21		
Range of score	1–3		
Practice			
Yes	0	< 0.0001	99.42–100.0
No	945 (100.0)		

Values have been represented as frequency (%) or mean ± standard deviation

Association of cumulative knowledge scores of the participants with their socio-demographic profile is summarized in Table 3. Of the 945 women who had answered these questions, women with inadequate knowledge were more likely to be those aged > 31 years (odds ratio (OR) = 1.94, 95% CI 1.40–2.70), widowed or separated women (OR = 3.96, 95% CI 1.65–8.99) and women with no primary level of education (OR = 19.10, 95% CI 11.4–32.06). Similarly, a significant association was observed between knowledge inadequacy and low socio-economic status (OR = 4.53, 95% CI 3.24–6.33) and Koraga and Malekudiya tribal community (OR = 3.25, 95% CI 2.24–4.71). Interestingly, women who were employed showed about 0.4 times lesser knowledge score than home-makers (OR = 0.59, 95% CI 0.43–0.80).

**Table 3:** Association of knowledge adequacy with the socio-demographic profile of the study population (*n* = 945)

Socio-demographic characteristics	Frequency (%)		OR (95% CI)			
	Knowledge inadequate (*n* = 733)	Knowledge adequate (*n* = 212)	Crude OR	*p* value	Adjusted OR	*p* value
Age (in years)						
≤ 30 (*n* = 247)	169 (68.4)	78 (31.6)	1.00	< 0.0001	1.00	0.14
> 30 (*n* = 698)	564 (80.8)	134 (19.2)	1.94 (1.40–2.70)		1.34 (0.91–1.98)	
Marital status						
Married (*n* = 865)	659 (76.2)	206 (23.8)	1.00	0.001	1.00	0.26
Widowed/separated (*n* = 80)	74 (92.5)	6 (7.5)	3.86 (1.65–8.99)		1.70 (0.67–4.34)	
Years of schooling						
Nil to primary level (*n* = 475)	458 (96.4)	17 (3.6)	19.10 (11.4–32.06)	< 0.0001	15.49 (9.10–26.36)	< 0.0001
Middle school and above (*n* = 470)	275 (58.5)	195 (41.5)	1.00		1.00	
Occupation						
Employed (*n* = 429)	354 (82.5)	75 (17.5)	1.00	0.001	1.00	0.49
Home-maker (*n* = 516)	379 (73.4)	137 (26.6)	0.59 (0.43–0.80)		1.14 (0.78–1.67)	
Socio-economic status						
Low (*n* = 535)	474 (88.6)	61 (11.4)	4.53 (3.24–6.33)	< 0.0001	1.81 (1.22–2.68)	0.003
Medium (*n* = 410)	259 (63.2)	151 (36.8)	1.00		1.00	
Tribal community						
Koraga and Malekudiya (*n* = 362)	321 (88.7)	41 (11.3)	3.25 (2.24–4.71)	< 0.0001	2.14 (1.37–3.35)	0.001
Marathi Naika (*n* = 583)	412 (70.7)	171 (29.3)	1.00		1.00	

Multivariable logistic regression analysis mutually adjusted for all the six socio-demographic variables showed that women with low level of education (AOR = 15.49, 95% CI 9.10–26.36), low socio-economic status (AOR = 1.81; 95% CI 1.22–2.68) and those belonging to Koraga and Malekudiya communities were more likely to be lacking knowledge about cervical cancer, its prevention and early detection.

## Discussion

Our study population had a suboptimal level of knowledge regarding cervical cancer and its prevention. Encouragingly, they showed a favourable attitude towards cancer screening and acceptance towards cancer education.

In this study, 82.9% participants had heard of cervical cancer which is similar to a report from Qatar (85.0%) and Cambodia (74%) and higher than that reported in Korea, Nepal and India where it ranged between 60 and 66% [14–18]. Although a little more than half of the study population (51.0%) knew that cervical cancer is preventable, only 2.3% of them were aware that the disease can be detected at an early stage. This is in contrast to the findings reported from Delhi (India), Ethiopia and Zimbabwe in which a higher level of knowledge was exhibited by the women [19–21]. This could be attributed to the difference in the nature of population studied and study settings. However, the results of our study are in agreement with that of previous studies from India in which majority (81.9–96.5%) of the women had poor knowledge [22–25]. As per previous published literature, lack of knowledge among women in the developing countries towards cervical cancer is mostly attributed to the paucity of organized cancer screening programs, sociocultural barriers and inefficient media campaigns in creating awareness [26].

In the present study, poor genital hygiene (38.2%) and early age at sexual intercourse (26.7%) were the most commonly quoted risk factors. However, in a study conducted in rural Kerala by Aswathi et al., a very small proportion of women were aware that poor genital hygiene (3.9%) and having multiple sexual partners (1.6%) are risk factors for cervical cancer [27]. In our study, knowledge regarding etiological role of viral infections in cervical cancer was very dismal (7.0%) compared with that of a study done by Arunadevi et al. in Tamil Nadu which reported a slightly higher proportion (13%) of women being aware about it [28].

Almost half of our study population (49.4%) were unaware about any of the risk factors for cervical cancer, while only 27.9% of the women in a study conducted in Cambodia, and 11% in another Indian study did not know about the risk factors [15, 28]. This difference could be due to low literacy and ignorance which were highly prevalent among our study population.

On assessing their attitude towards prevention of cancer cervix, majority (> 90%) of tribal women exhibited favourable attitude which is in contrast to the findings reported in previous Indian studies from Bhopal conducted by Bansal et al. (80.5%) and in Andhra Pradesh by Narayana G et al. (62.5%), though the way of assessment of attitudes varied widely [29, 30].

None of our study population had ever undergone screening test for cancer cervix prior to the survey. However, many community-based studies from Cambodia, Nepal and Ethiopia [15, 17, 18] reported a small proportion of women being screened for cancer cervix with Pap test (ranged from 7.1 to 13.6%) and so did the previously published Indian studies (6.9–13.4%) [18, 27, 30]. Poor cancer screening practice among the study population could be explained by their poor health-seeking behaviour and non-availability of organized cancer screening facilities in remote areas.

In the present study, predominant source of information regarding cervical cancer was family and friends (97.1%) followed by mass media (65.8%) and healthcare workers (23%). On the other hand, media was reported as the most common source of information on cervical cancer by many of the earlier studies [15, 28, 30, 31]. It was worthwhile to observe that over 88% of the participants thought cervical cancer screening was important and over 99% opined that regular screening services should be made available in their areas. Moreover, women were found to have favourable attitude despite the poor knowledge and practices towards cervical cancer prevention in our study. This finding is consistent with the result of studies done by Bansal et al. from Bhopal and Shreshta et al. and Thapa et al. from Nepal [17, 29, 32].

On assessing the association of cumulative knowledge score with age of the study population, younger women (30 years or less) were observed to have higher scores which is in contrast to the previously published studies where direct proportionality of age with better knowledge was reported [17–19]. The higher levels of knowledge among the participants with younger age could be due to the availability and ease of accessibility of information in this era of information technology. Women with higher literacy level were found to be significantly associated with better knowledge which is similar to the findings of previously published studies [14, 17]. Women who were employed were observed to lack adequate knowledge than those who were home-makers. This contradicts the findings of a study conducted by Al-Meer et al. in Qatar [14]. Since no significant difference was observed in the source of information among employed and home-makers, the data warrants further investigation. Women with better socio-economic status displayed better knowledge scores in the study. This is in concordance with the study by Narayan et al., wherein participants with low household income exhibited poor knowledge and unfavourable attitude towards cervical cancer screening [30]. Moreover, knowledge scores among women from the Koraga and Malekudiya tribal communities combined were significantly poorer as compared with those from Marathi Naika community. This could be due to the fact that people from Marathi Naika community have been receiving better formal education and employment in recent years and progressing towards the mainstream population.

This is a first of its kind community-based survey, to the best of authors’ knowledge, conducted to study the KAP towards cervical cancer among tribal women in the coastal region of southern India. This data provides pertinent information for designing appropriate interventions to improve cervical cancer awareness and screening practices among the vulnerable populations. However, due to the cross-sectional nature of our study, we might not have been able to elucidate all the determinants of KAP towards cervical cancer among these women.

## Conclusion

As observed in the study, there is a considerable lack of knowledge and practices towards cervical cancer and its screening among the tribal women of this region, though they had a favourable attitude. The study identified that being an illiterate or a school drop-out, having low socio-economic status and belonging to Koraga and Malekudiya tribal communities were the significant predictors for lack of knowledge towards cervical cancer among tribal women. Hence, there is a need for well-organized cancer education programs to create awareness and to eliminate misconceptions regarding prevention of cervical cancer in this community. In addition, provision of comprehensive cancer screening facilities at primary care level would improve the cervical cancer screening practices among these vulnerable populations. Further qualitative studies are essential to understand the prevailing barriers regarding cervical cancer screening among tribal women in this region.
